# Pathological Mobilization and Activities of Dendritic Cells in Tumor-Bearing Hosts: Challenges and Opportunities for Immunotherapy of Cancer

**DOI:** 10.3389/fimmu.2013.00435

**Published:** 2013-12-10

**Authors:** Amelia J. Tesone, Nikolaos Svoronos, Michael J. Allegrezza, Jose R. Conejo-Garcia

**Affiliations:** ^1^Tumor Microenvironment and Metastasis Program, Wistar Institute, Philadelphia, PA, USA; ^2^Cell and Molecular Biology, Perelman School of Medicine, University of Pennsylvania, Philadelphia, PA, USA

**Keywords:** myelopoiesis, dendritic cell, tumor microenvironment, immune suppression, *in situ* vaccination, cancer immunotherapy

## Abstract

A common characteristic of solid tumors is the pathological recruitment of immunosuppressive myeloid cells, which in certain tumors includes dendritic cells (DCs). DCs are of particular interest in the field of cancer immunotherapy because they induce potent and highly specific anti-tumor immune responses, particularly in the early phase of tumorigenesis. However, as tumors progress, these cells can be transformed into regulatory cells that contribute to an immunosuppressive microenvironment favoring tumor growth. Therefore, controlling DC phenotype has the potential to elicit effective anti-tumor responses while simultaneously weakening the tumor’s ability to protect itself from immune attack. This review focuses on the dual nature of DCs in the tumor microenvironment, the regulation of DC phenotype, and the prospect of modifying DCs *in situ* as a novel immunotherapeutic approach.

## Introduction

Accumulated experimental and clinical evidence indicate that the immune system recognizes neoplasms and attempts to mount a response against these altered cells. However, immune pressure against established tumors is clearly not sufficient to defend tumor-bearing hosts from disease progression, and eventually death. A universal occurrence in established tumor-bearing individuals is a profound alteration of myelopoiesis (1). Pathological myeloid differentiation leads to the expansion of a heterogeneous population of immunosuppressive myeloid cells that accumulates in the spleen and gives rise to regulatory macrophages and dendritic cells (DCs) in tumors (2). This diverse mix of pathological myeloid cells at different stages of differentiation (generically termed Myeloid-Derived Suppressor Cells, or MDSCs) is highly immunosuppressive (1, 3). MDSCs also contribute to enhanced angiogenesis (4), as well as the formation of metastatic niches for malignant dissemination (5, 6). Additionally, defective development alters the critical function of myeloid cells that, under normal physiological conditions, would terminally differentiate into DCs, macrophages, or neutrophils. Defective myleopoiesis results in a significant defect in antigen presentation, which is aggravated during malignant progression, and drives T cell-intrinsic transcriptional programs that promote T cell anergy and exhaustion. In contrast, certain tumors mobilize excessive amounts of lineage-committed, classical CD11c^+^ DCs that, rather than driving tumor antigen-specific responses, impair T cell effector function at the tumor bed. Here, we will review how pathological myelopoiesis and tumor microenvironmental networks progressively abrogate the immunostimulatory function of DCs, resulting in unresponsive T cells and prevention of the lingering immune pressure exerted by remaining tumor-reactive lymphocytes. We will conclude by discussing potential approaches to overcome these effects *in vivo* and *in situ*.

## Tumors Promote Defective DC Differentiation and Maturation

Dendritic cells originate in the bone marrow from the differentiation of hematopoietic precursors to Common Myeloid Progenitors and subsequently to DCs. Recent evidence indicates that, at least in mice, precursors of conventional DCs specifically express Clec9a and represent an independent lineage much less dependent on inflammation-induced monocyte differentiation than previously thought (7).

**Figure 1:**
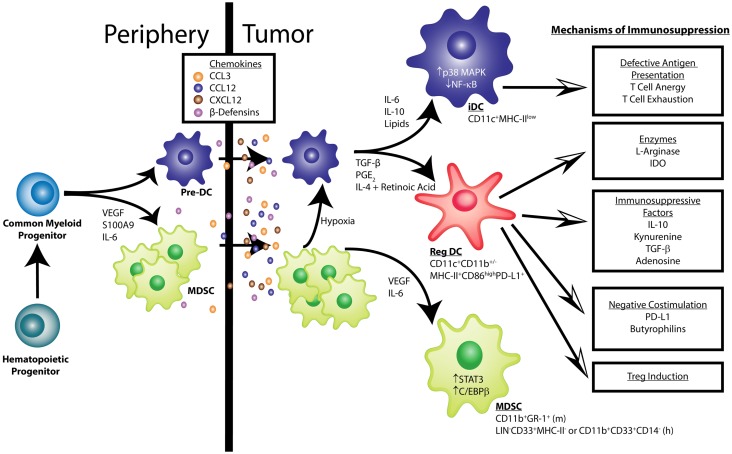
**Pathological dendritic cell differentiation contributes to tumor-induced immune evasion**. Cancer-associated inflammation up-regulates the production of myeloid cells from hematopoietic progenitors in the bone marrow. Common myeloid progenitor cells give rise to pre-dendritic cells (pre-DCs) and, under the influence of tumor-derived factors (e.g., VEGF, IL-6, and S100A9), myeloid-derived suppressor cells (MDSCs). These myeloid cells migrate into the tumor microenvironment in response to chemokines such as CCL3, CCL12, CXCL12, and β-Defensins. VEGF and IL-6 activate STAT3 and C/EBPβ signaling in MDSCs, keeping them in an immature phenotype characterized as CD11b^+^Gr-1^+^in mice and LIN^−^CD33^+^MHC-II^−^or CD11b^+^CD33^+^CD14^−^ in humans. Additionally, IL-6, IL-10, and the accumulation of lipids can activate p38 MAPK signaling to prevent the acquisition of classic DC function. Also, some MDSCs can differentiate into DCs in the hypoxic environment of the tumor. Immature DCs (iDCs) exhibit low NF-κB activation, express CD11c in mice and humans, and have low MHC-II levels. These iDCs are defective antigen presenters, which induces T cell anergy and exhaustion. In other conditions, like those found in epithelial ovarian cancer, pre-DCs mature into cells that express markers of conventional DCs (CD11c^+^ MHC-II^+^CD86^high^), but exert immunosuppressive functions, termed regulatory DCs (Reg DCs). Factors such as TGF-β, PGE_2_, IL-4, and retinoic acid have been shown to promote this altered maturation. These Reg DCs differ from conventional DCs in their ability to suppress effector T cell function through multiple mechanisms, which include: (1) secretion of the enzymes l-Arginase and IDO that result in the depletion of essential amino acids and production of the tolerogenic metabolites adenosine and kynurenine; (2) release of immunosuppressive factors such as IL-10 and TGF-β; (3) expression of costimulatory surface molecules, including PD-L1 and butyrophilins, that negatively regulate anti-tumor T cells; (4) induction of regulatory T cells (Tregs). More information regarding the source of the various secreted factors that govern the accumulation and function of tumor-associated myeloid cells can be found in recent reviews by Hanahan and Weinberg as well as Lindau et al. (17, 18).

A hallmark of virtually all solid tumors is aberrant expansion of pathologically differentiated myeloid leukocytes (see Table 1). These cells arise from myeloid progenitors under the influence of inflammatory signals (8) and accumulate at splenic, lymphatic, and tumor locations. While they retain an immature phenotype, these MDSCs are highly immunosuppressive (9). Elegant experiments based on transfer of tumor-derived MDSCs of the myelomonocytic lineage have shown that under the influence of hypoxia in the tumor microenvironment (TME), these altered precursors of macrophages and DCs still reach their terminally differentiated cell fates (2). However, as expected from the succession of non-physiological signals that these myeloid cells receive, their phenotype is quite different from canonical macrophages and DCs generated under steady state conditions (see Figure 1). Tumor-differentiated macrophages, for instance, retain an immunosuppressive phenotype that contributes to accelerate malignant progression.

**Table 1:** **Phenotypic features of different tumor-infiltrating myeloid cell populations**.

Cell type	Other names	Surface markers	Chemokine receptors	Phenotype	Human tumors observed within	Reference
MDSC		CD11b^+^GR-1^+^ (m), Lin-CD33^+^MHC-II^−^or CD11b^+^CD33^+^CD14^−^(h)		Immunosuppressive	Breast, renal-cell, pancreatic, melanoma, head and neck	Gabrilovich and Nagaraj (9)
Mature DC	Classical DC	CD11c^+^MHC-II^high^	CCR7, CXCR4	Immunostimulatory		
Immature DC		CD11c^+^MHC-II^low^	CCR6, CCR2, CXCR4	Antigen uptake, Immunosuppression	Ovarian, breast, lung, colorectal, melanoma, renal-cell, prostate	Chaux et al. (10), Pinzon-Charry et al. (11), Perrot et al. (12)
Pre-DC		CD11c^+^MHC-II^−^	CCR1, CCR5, CCR2	Committed to DC lineage		
Regulatory DC	Tolerogenic DC	CD11c^+^CD11b^±^, MHC-II^+^CD86^high^PD-L1^+^	CXCR4, CCR6	Immunosuppressive	Cervical, hepatocellular, breast, ovarian	Lee et al. (13), Ormandy et al. (14), Pinzon-Charry et al.(15), Scarlett et al. (16)

*Mouse and human markers are indicated by (m) and (h), respectively*.

In dissecting the role of DCs in cancer, most studies have focused on impaired DC differentiation as the cause of diminished production of mature, functionally competent DCs (1, 19). In support of this concept, a decrease in the accumulation of mature DCs has been found in patients with cervical (13), hepatocellular carcinoma (14), lung (12), colorectal (10), and breast cancer (15). Blockade of DC differentiation in tumor-bearing hosts was primarily attributed to VEGF, a common tumor microenvironmental factor widely known for its role in promoting tumor angiogenesis (20, 21). Accordingly, VEGF levels negatively correlate with the number of DCs in the blood or tumor in a variety of human cancers (22–24). These studies implied that the paucity of functionally mature DCs in tumors was a major contributing mechanism to overall immune evasion.

The consequence of defective antigen presentation in cancer individuals is T cell unresponsiveness, which may be the result of anergy or exhaustion. Unlike replicative senescence, anergy and exhaustion are reversible processes that result from different transcriptional programs but are frequently confused (25, 26). Anergy takes place at the time of priming, while exhaustion occurs in previously activated T cells undergoing repeated exposure to suboptimal amounts of antigen in the presence of negative costimulation. As emerging clinical evidence using PD-1 inhibitors suggests, T cell exhaustion is a major driver of tumor-induced immunosuppression in more than a third of cancer patients (27, 28).

## Tumors also Induce the Accumulation of Fully Committed DCs with Immunosuppressive Activity

While impaired DC differentiation and maturation may explain the myeloid phenotypes found in some tumors, they do not explain the accumulation of DCs with regulatory phenotypes that we and others have found in certain tumors (16, 29). Thus, we showed that classical DCs with immunosuppressive activity, termed regulatory DCs, accumulate in the TME. In tumors, these DCs suppress T cell effector functions through multiple mechanisms that include the expression of PD-L1, the production of l-Arginase and the up-regulation of tolerogenic butyrophilins (16, 30–38). The contribution of classical DCs to tumor-induced immunosuppression is therefore different from the mere lack of fully differentiated, immunostimulatory DCs, at least in certain carcinomas (see Table 1).

Because the myeloid leukocytes that are found in the TME represent a heterogeneous mix of abnormal cells at different stages of differentiation, phenotypic overlap and variability between patients hinder a conclusive categorization of macrophages vs. differentiated DCs vs. more immature precursors across tumor specimens. Nevertheless, we have demonstrated that the predominant population infiltrating solid human ovarian cancer specimens (but not human ascites) exhibits predominant determinants of DCs, including CD11c, HLA-DR, and DEC205, but do not express the monocyte/macrophage markers CD11b or CD14 in at least 1/3 of patients (16). Irrespective of nomenclature in human tumors, we have also demonstrated that the counterpart of this population in ovarian cancer mouse models can be induced to process full-length OVA *in vitro* (30) and *in vivo* (32, 34), and effectively present processed SIINFEKL to T cells in response to certain activating signals. These CD11c^+^ cells also produce Zbtb46 transcripts (39, 40) and express Clec9a (7) further implying their DC nature. DCs are therefore important players of the immunosuppressive networks orchestrated by at least some frequent epithelial tumors, and defective antigen-presenting activity contributes to the abrogation of the protective function of anti-tumor T cells.

We initially assumed that these DCs were “immature,” and therefore simply unable to prime T cell responses. However, ovarian cancer DCs express significant levels of CD86. Even more surprisingly, human tumor DCs in multiple specimens also express CD83, an activation marker. Furthermore, these DCs produce high levels of inflammatory cytokines such as IL-6 and the chemokine CCL3 (32–34). Additionally, although TLR activation can further up-regulate MHC-II, these DCs express relatively high MHC-II levels in the TME, in both humans and mice (16, 37). Most importantly, progressive weakening of anti-tumor immunity cannot be solely attributed to “scarcity” of mature DCs in these tumors because depleting DCs at advanced stages of malignant progression in preclinical models paradoxically delays tumor growth, rather than being simply “neutral” (16). Excessive accumulation of immunosuppressive DCs, rather than mere absence of immunostimulatory antigen-presenting cells (APCs), is therefore the predominant mechanism of DC dysregulation in at least ovarian carcinoma.

These regulatory DCs are also different than their immature precursors due to their main location of action. Immature DCs that fail to efficiently activate T cells in the lymph node will primarily prevent T cell priming, leading to anergy or tolerization. And while we have identified immunosuppressive, regulatory DCs in the draining lymph node (16), the remarkable suppression by tumor-infiltrating DCs contributes to a protective barrier for tumor cell growth. By suppressing effector T cells through many mechanisms we discuss here, tumor-infiltrating DCs can effectively shut down activated anti-tumor immune responses. This important difference has imperative consequences for the fate of therapies that rely solely on eliciting tumor-directed T cells. For this reason, we mostly focus on the action of these altered DCs inside the tumor in this review.

Based on our converging evidence, we propose that regulatory DCs in tumors are not immature, but acquire an alternative phenotype in response to a different transcriptional program. Some peculiarities of this aberrant program have been identified in our recent studies. Thus, we demonstrated that delivery of synthetic (and functional) microRNA-155 (miR-155) specifically to CD11c^+^DEC205^+^MHC-II^+^ DCs in the microenvironment of ovarian cancer-bearing mice induced genome-wide transcriptional changes that were sufficient to transform these immunosuppressive leukocytes into an immunostimulatory cell type (37). Compared to the delivery of non-targeting double-stranded RNA of similar length and structure, ectopic miR-155 induced silencing of multiple immunosuppressive mediators, including *Tgfb1* and *Cd200*. As expected, miR-155 also targeted crucial transcription factors involved in aberrant myeloid differentiation in tumor-bearing hosts, including *Cepb/*β and *Socs1*. Most importantly, miR-155 activity down-regulated *Stat3* and, interestingly, *Satb1*, a master genomic organizer (37, 41). Unexpectedly, however, expression of miR-155 also silenced the expression of *Pu.1/Sfpi1* and *Irf8* (>150-fold in both cases) (37). This is significant because PU.1 promotes DC differentiation by inducing the remodeling of the higher-order chromatin structure at the *Irf8* gene. Therefore, myeloid IRF8 expression depends on high PU.1 levels (42). Most importantly, DC commitment requires active IRF8 to avoid alternative myeloid-lineage differentiation (42). The fact that synthetic miR-155-expressing DCs acquire the capacity to effectively present antigens *in vivo* and *in vitro* suggests that they were fully committed to a DC phenotype, albeit transcriptionally different from their classical immunostimulatory counterparts.

## Tumor- and Stroma-Derived Chemoattractants Drive DC Recruitment to Tumor Locations

The presence of regulatory DCs, as opposed to simply immature precursors, in the microenvironment of different tumors can be partially driven by the abundance and repertoire of tumor- and stroma-derived chemokines and cytokines, which is dependent on differential DC expression of chemokine receptors (43–45). For instance, the chemokine CCL3, aberrantly up-regulated in many tumors, enhances pre-DC recruitment in both *in vitro* and *in vivo* models of melanoma, colon, and lung carcinoma. Moreover, CCL3 preferentially recruits pre-DCs to tumor locations, as antibody mediated neutralization of CCL3 does not result in a decrease in any other CD45^+^ leukocyte subset, nor does it alter the frequency of splenic pre-DCs (46). Furthermore, pre-DC expression of CCR1 and CCR5 provide a mechanism for CCL3-mediated recruitment (47). In addition, tumor hypoxia, which characterizes a wide variety of TMEs, also triggers DC and immature myeloid cell recruitment via tumor-derived CCL12.

Additionally, tumor microenvironmental prostaglandin E_2_ (PGE_2_) up-regulates the chemokine CXCL12, thereby accumulating CXCR4-expressing regulatory DCs (48, 49). Another relatively uninvestigated factor that promotes the recruitment of CCR6^+^ regulatory DCs to tumor locations is the expression of β-Defensins by epithelial cells and inflammatory leukocytes. In preclinical models, ectopic tumor expression of *Defb29* has been shown to accelerate the accumulation of DCs in the TME, leading to more aggressive malignant progression (30). Defensins are clearly powerful chemoattractants (50) that are expressed at significantly higher levels in certain cancer patients (51).

Perhaps the most compelling evidence that tumor-derived chemokines influence the type of DCs found in the tumor has been demonstrated in a renal-cell carcinoma model (52), where the intra-tumoral or peri-tumoral distribution of DCs is determined by CCL20 and CCL19 levels. High intra-tumoral levels of CCL20 result in the accumulation of immature CCR6^+^ DCs within the tumor bed, whereas high peri-tumoral levels of CCL19 promote the accumulation of mature CCR7^+^ DCs preferentially to the tumor margin.

## Pathways Preventing Dendritic Cell Differentiation

The accumulation of immature DCs and earlier progenitors are also the result of corrupted pathways of inflammation-driven myeloid differentiation. The extent of the corrupted nature of myelopoiesis in cancer has been underscored by recent studies by Gabrilovich and colleagues showing that the epigenetic silencing of the retinoblastoma gene in immature (non-tumor) myeloid cells is sufficient to drive transdifferentiation of myelomonocytic MDSCs to granulocytic MDSCs (53). A block in the differentiation of immature myeloid precursors into mature myeloid cells results in fewer DCs and an accumulation of MDSCs. The molecular pathways driving this process are known to be STAT3-dependent. For instance, high STAT3 activity has been shown to inhibit both the differentiation of mature DCs from myeloid precursors (54) and the activation and MHC-II up-regulation of DCs (55). Still, the molecular mechanism of this STAT3 signaling remains incompletely understood. Studies by Lee and colleagues have shown that tumors mediate both STAT3 activation and PKC βII down-regulation in DC progenitor cells (56). Importantly, PKC βII repression can be mimicked by the expression of a constitutively active STAT3 mutant. Because PKC βII is required for DC differentiation (57), these reports have identified a roadblock for subsequent maturation of myeloid precursors, but more studies are needed to fully dissect this mechanism.

Many factors overexpressed in the tumor macro- and microenvironments promote STAT3 activation. It has been demonstrated that the negative effect of tumor conditioned media on DC activation could be reversed by using either STAT3 null DCs or DCs treated with a peptide inhibitor of STAT3 (58). Some mediators of this process include IL-6, IL-10, and VEGF, which are generally increased systemically in cancer patients and negatively correlate with prognosis (59). The STAT3 activator IL-6 is particularly important for the development of functionally defective DCs (60). Tumor-derived IL-6 drives STAT3 nuclear translocation in immature myeloid cells, thus promoting proliferation and inhibiting apoptosis of MDSCs. Furthermore, IL-6 and M-CSF secreted by human renal carcinoma cell lines inhibit DC differentiation from CD34^+^ bone marrow progenitors (61). In addition, STAT3 up-regulates S100A9 in hematopoietic progenitor cells, which inhibits further differentiation to DCs and retains these cells as MDSCs, leading to tumor tolerance (62).

Besides STAT3, some of these cytokines inducing MDSCs also act on a common molecular pathway that is entirely dependent on the C/EBPβ transcription factor. C/EBPβ is therefore required for the immunoregulatory activity of both tumor-induced and bone marrow-derived MDSCs (63).

Additionally, VEGF has been shown to impair DC differentiation from progenitors through the inhibition of another pathway that is also important for DC maturation; namely, by preventing NF-κB activation (21, 64).

## Pathways Preventing Dendritic Cell Maturation

Besides impairment of DC development, DCs can be found in an active immature phenotype associated with induction of peripheral tolerance. Numerous studies have demonstrated that these immature DCs (iDCs) are unable to support normal levels of antigen-specific T cell expansion. For example, human monocyte-derived DCs (mo-DCs) are unable to induce expansion of antigen-specific, tetramer-positive T cells when kept in an immature phenotype by differentiation in the presence of the adenosine receptor agonist NECA or cAMP elevating agents FSK/IBMX (65).

Under steady state conditions, DCs exist in an immature state characterized by high phagocytic activity and low antigen-presenting capabilities. Upon being activated by innate pathogen or damage associated signals, DCs acquire a mature state characterized by MHC presentation of antigens and costimulatory signaling via B7 family molecules (e.g., CD80 and CD86). For an immature DC to become an efficient APC, it must receive specific activating signals through receptors such as CD40, TNF-R, IL-1R, and TLRs (66). Although the intricacies of how DC maturation is blocked remain to be fully dissected, an important mechanism preventing DC maturation appears to be mediated by the p38 MAPK pathway. IL-6, IL-10, and TGF-β contained in myeloma-conditioned media have been shown to activate this pathway and prevent the immunostimulatory activity of immature DCs. Abrogating p38 MAPK with a small molecule inhibitor during DC development enhanced their subsequent activation, making them more mature and stimulatory even when differentiated in the conditioned media (67). In addition, paclitaxel-induced inhibition of p38 MAPK activity decreases the production of S100A9 and TNF-α by MDSCs, resulting in reduced tumor burden and increased animal survival (68). In tumor-bearing mice, paclitaxel induces the maturation-dependent antigen-presenting activity of DCs (69).

High levels of IL-10 in tumor-bearing hosts also impair the complete maturation of DCs. DCs from mice that overexpress IL-10 have low expression of MHC-I and costimulatory molecules and are deficient at stimulating T cell responses (70). Accordingly, IL-10 treated human DCs induce anergy in T cells, although fully matured DCs were resistant to the effect of IL-10 (71). Pancreatic tumor cells were also found to secrete IL-6 and IL-10 thus suppressing the stimulatory abilities of DCs in allogeneic reactions (72).

Another pathway relevant for effective DC maturation is dependent on NF-κB activation. Signaling via NF-κB is required for the professional APC function of DCs, including high expression of MHC-II and costimulatory molecules, secretion of IL-12 and TNFα, and stimulation of allogeneic T cells (73). Recently, PD-1 expression on DCs from murine ovarian tumors was found to decrease NF-κB activation, resulting in less production of inflammatory cytokines (74).

Additional mechanisms that remain poorly understood but could be very important for preventing the immunostimulatory potential of tumor-associated DCs are dependent on how myeloid leukocytes metabolize lipids in the TME. Tumor DCs up-regulate scavenger receptor A (SR-A), a target used for therapeutic depletion of regulatory DCs in preclinical models (75). Overexpression of SR-A increases the uptake of extracellular lipids, which are pathologically accumulated in DCs (76). Tumor DCs from mice and humans were demonstrated to have relatively high levels of triglycerides that impaired their ability to process antigen, and could be functionally restored upon normalization of their triglyceride levels.

Finally, immunomodulatory signals can also come from metabolites, as tumor-derived lactate can skew the differentiation of human monocytes into less mature DCs (defined by less CD1a expression) that are deficient in their ability to stimulate T cells and secrete IL-12 (77).

## Pathways Driving the Transformation of Dendritic Cells into an Immunosuppressive Cell Type

In addition to blocking DC differentiation and full activation, the TME also contains multiple factors that transform classical DCs with antigen-presenting capabilities into immunosuppressive players. Our recent studies in a preclinical model of sarcomatoid carcinoma in immunocompetent mice have illustrated that this switch takes place during malignant progression (16, 37). In this system, the inflammatory microenvironment of advanced tumors recapitulates the molecular and cellular components of immune cells found in human solid tumors. We found that at initial stages of tumor development, DCs elicit T cell responses that are able to put tumor growth in check for relatively long periods. As expected, depleting DCs at early stages of tumor progression resulted in accelerated tumor progression, which mimicked T cell depletion. However, as these tumors advanced, DC differentiation was not blocked. Rather, CD11c^+^MHC-II^+^DEC205^+^ DCs with regulatory activity started accumulating at tumor locations, which coincided with the beginning of the exponential growth phase of these latent tumors. Strikingly, DC depletion at this advanced stage of malignant growth was sufficient to significantly delay (rather than advance) malignant progression. DCs in these late-stage tumors are therefore not simply immature or unable to effectively present antigens. In fact, although DCs in advanced tumors exhibited lower expression of MHC-II and costimulatory CD40, they still expressed both at significant levels. Instead they became active accomplices to tumor growth through the inhibition of protective immunity.

Dendritic cell accumulation in solid tumors is not only found in the microenvironment of solid ovarian carcinomas. For instance, Norian et al. found that MHC-II^+^CD11b^+^CD11c^high^ DCs infiltrating established mammary carcinomas could also act as regulatory players by inhibiting CD8 T cell function through l-Arginase production, thus dampening T cell-mediated anti-tumor immune protection (29). Naïve T cells primed with these DCs undergo minimal expansion and defective IFN-γ production, and eventually become anergic. The phenotype of these regulatory DCs therefore does not correspond to the plasmacytoid DC type that has been traditionally associated with the development and maintenance of immunosuppression, although these cells are also found in the microenvironment of many tumors (78–81).

Among the potential tumor microenvironmental factors driving the transformation of immunostimulatory DCs into immunosuppressive players, we identified that at least PGE_2_ and TGF-β (but not IL-6) in supernatants from primary cultures, are both necessary to elicit a regulatory phenotypic switch in DCs from early tumors. These DCs were then capable of suppressing the strong proliferation of tumor-reactive T cells in response to tumor antigen presented by other DCs (16, 37).

In addition, IL-4 and retinoic acid synergize to induce the expression of *Aldh1a2* in GM-CSF-differentiated inflammatory DCs, turning on their regulatory activity (82). Retinoic acid is also known to enhance TGF-β-induced Smad3 activation (83), potentially synergizing with the induction of suppressive features elicited by TGF-β on DCs (16, 37).

Another important factor dampening the immunostimulatory potential of tumor DCs is kynurenine, the first product of the tryptophan degradation pathway generated by Indoleamine-pyrrole 2,3-dioxygenase (IDO) (84, 85). Kynurenine, by interacting with the aryl hydrocarbon receptor (AHR), elicits an autocrine loop in DCs, resulting in enhanced IDO activity and the acquisition of an immunosuppressive phenotype by originally immunocompetent DCs (86). In another study, IDO expression was found to be required for DC-induced tolerance, and, via TGF-β-induced expression of IDO, it was possible to convert CD8-negative DCs from being immunogenic to regulatory (87).

## Mechanisms of Immunosuppression Driven by DC Secretion of Immunosuppressive Factors

Regulatory DCs suppress immune responses by secreting anti-inflammatory soluble factors that inhibit effector T cell functions or skew T cell responses. DCs, for instance, are a major source of IDO within the TME. DCs infiltrating multiple tumors show enhanced IDO activity. For example, IDO-expressing FOXO3^+^ DCs were shown to promote malignant progression in preclinical models of prostate cancer (88). Kynurenine produced by IDO activates AHR, which is central to T cell differentiation into FoxP3^+^ regulatory T cells (Tregs) (89, 90). Generation of induced Tregs is not only a property of murine tumor DCs, as IDO expression in human DCs also results in induction of Foxp3^+^, immunosuppressive T cells when these DCs are co-cultured with healthy donor CD3^+^ lymphocytes. This induction was confirmed to be IDO-dependent by reversal of T cell phenotype following tryptophan treatment (91).

Regulatory DCs, and not only macrophages or MDSCs, are also important contributors to immunosuppression in the TME through the production of l-Arginase (16, 29, 37). l-Arginase activity results in catabolic depletion of Arginine, an amino acid essential for effector T cells. Upon undergoing a phenotypic switch from being immunostimulatory to immunosuppressive, DCs found in advanced tumors significantly increase l-Arginase activity. Importantly, freshly dissociated human ovarian carcinoma specimens also contain DCs with significant l-Arginase activity (16, 37).

Among the factors that enhance l-Arginase activity in the TME, IL-6 is perhaps the best characterized. IL-6 treatment of bone marrow-derived DCs (BMDCs) results in increased l-Arginase RNA, protein, and activity both *in vitro* and *in vivo*. The consequent drop in extracellular arginine resulted in down-regulation of MHC-II in the same DCs and impaired ability to activate OT-II T cells. These results were similarly reproduced by differentiating BMDCs in arginine-free conditions (92).

Other immunosuppressive factors produced by DCs in advanced tumors include cytokines such as TGF-β and IL-10. Tumors induce DCs to secrete TGF-β, further promoting Treg expansion and indirectly suppressing T cell effector functions (93, 94). However, the relative contribution of DC-derived TGF-β compared to the production of this cytokine by other microenvironmental cell types (e.g., certain T cell subsets, including Tregs), needs to be comprehensively addressed.

## Mechanisms of Immunosuppression Driven by DCs through Membrane-Bound Determinants

In addition to not providing sufficient antigen or costimulation, tumor-associated DCs frequently express negative costimulatory molecules that suppress T cell activity. As evidenced by the emerging success of novel clinical inhibitors, perhaps the most important negative signaling is mediated through PD-L1:PD-1 interactions (95). PD-1 is a negative costimulatory receptor primarily expressed on activated lymphocytes. The most abundant ligand for PD-1, B7-H1/PD-L1, is up-regulated in DCs and tumor cells in multiple cancers. PD-1 itself can also be expressed by tumor DCs themselves, at least in murine models. Expression of the PD-L1:PD-1 pair was found to increase throughout malignant progression, correlating with loss of positive costimulatory markers (CD80, CD86, and CD40), a lack of cytokine release (IL-12, IL-10, IL-6, TNFα, and G-CSF), and contact-dependent inhibition of T cell expansion (74). Importantly, inhibitors for both the ligand (PD-L1) and the receptor (PD-1) have been developed and have shown impressive clinical results (27, 28). However, PD-1 blockers appear to be better candidates for future FDA approval, while PD-L1 inhibitors appear to produce better results in murine models (96), possibly related to the affinity and pharmacokinetics of different humanized and mouse-specific antibodies.

Aside from PD-L1, regulatory DCs can express other negative costimulatory molecules. One of these inhibitors, universally expressed in ovarian cancer-infiltrating DCs, is CD277 (35). CD277 identifies various highly similar members of the butyrophilin subfamily 3 (BTN3). The function of these molecules is poorly investigated, but they share sequence and structural homology to the negative costimulatory molecule B7-H4. CD277 is expressed by CD45^+^ MHC-II^+^ APCs isolated from human epithelial ovarian cancer samples, and is up-regulated in human mo-DCs in response to molecules found in the TME, such as IL-6, IL-10, VEGF, PIGF-1, and CCL3. We showed that CD277 expressed in artificial APCs consistently decreased the expansion of TCR-stimulated T cells. However, one of the butyrophilins expressing the CD277 epitope (BTN3A1), has been recently reported as an activating receptor (thus not a ligand) in γδ T cells, where it binds phosphorylated antigens with low affinity (97). In addition, other similar molecules such as BTNL8 have been associated with immunostimulatory activity when a soluble fusion protein was used (98). It is unclear, however, whether the activity of butyrophilins depends on engagement of the unknown receptor in T cells in a cross-linked or soluble form.

Also important for the generation of tolerogenic mediators are the two ecto-enzymes CD39 and CD73 that act sequentially to generate anti-inflammatory extracellular adenosine (72, 99, 100). Among the many suppression-promoting effects that TGF-β induces in DCs, it up-regulates the expression of CD73 (101). CD73 produces adenosine from AMP, which engages with the adenosine A2A receptor (A2AR). A2AR ligation both inhibits the expansion of effector T cells and promotes the generation of induced Tregs (102). Chemical enzymatic inhibitors or neutralizing antibodies targeting these ecto-enzymes therefore offer novel promising avenues of therapeutic interventions.

## Implications of DC Dysregulation in Cancer Patients for Therapeutic Dendritic Cell-Based Vaccines

Because DCs are the most potent APCs, they have been used to boost T cell-mediated immune protection against cancer for nearly two decades. Multiple approaches have been shown to work effectively in mice, primarily as prophylactic interventions. However, any reproducible clinical benefit for patients with established cancer has been marginal so far (103). It is true that there is still room for improvement, especially regarding immunostimulatory cell type and route of administration. However, converging clinical evidence suggests that quantifiable improvements in immunological readouts are not associated with reproducible clinical responses (104).

Although DC-based vaccines are designed to overcome defective maturation, challenges with migration to places of T cell priming and, especially, the abrasive effect of the immunosuppressive networks in the TME, have so far rendered vaccine-induced T cell responses ineffective against tumor-induced tolerance. A successful trial using autologous lysate-pulsed DC vaccination in recurrent ovarian cancer has become an auspicious exception (105). However, DC vaccination in this trial was followed by adoptive transfer of vaccine-primed, *ex vivo* stimulated T cells, and it is therefore unclear whether the obvious clinical responses can be at all attributed to the initial vaccine. Nevertheless, this approach opens new avenues for the use of DCs for effective priming of autologous tumor-reactive T cells *ex vivo*.

All the aforementioned mechanisms of DC dysfunction in advanced malignancy contribute to our understanding of the failure of DC vaccines to deliver on their original promise. Even if *ex vivo* matured DCs could reach lymph nodes and can effectively prime tumor antigen-specific T cells without being affected by TFG-β, retinoic acid, or IDO metabolites, it is becoming increasingly clear that anti-tumor T cells will succumb in the TME, unless immunosuppression is concurrently targeted. Consequently, new opportunities emerge from the use of DC-based vaccines to treat early-stage disease, where they can be more efficacious in the absence of systemic immune dysfunction (106). DC-based vaccines, for instance, are now being tested against ductal carcinoma *in situ* (DCIS), to prevent development of subsequent breast cancer. Recent trials observed reduced recurrences in patients with estrogen receptor negative DCIS. Because the immunosuppressive networks are not as strong at this disease stage, this approach may be more promising (107).

## Reversing the Phenotype of Dendritic Cells from an Immunosuppressive to an Immunostimulatory Cell Type

While the establishment of regulatory DCs is a significant pathological event in solid tumors, the central role that DCs play in orchestrating adaptive immunity still offers opportunities for therapeutic intervention that could have lasting benefit. *In situ* vaccine-based interventions aimed at transforming tumor DCs into activated APCs capable of priming host anti-tumor T cells represent a promising approach to both actively boost T cells and inhibit immunosuppression. Vicari and colleagues first reported that tumor-infiltrating DCs are able to be rescued and become effective tumor antigen presenters in the context of MHC-I, provided that they receive the right stimulatory signals. They found that DCs were refractory to stimulation with the combination of LPS, IFN-γ, and anti-CD40 antibody, but tumor-induced DC paralysis could be reverted by a combination of CpGs and an anti-IL-10R antibody (108).

The combination of signals promoting the immunostimulatory capacity of otherwise immunosuppressive DCs may depend on different tumor settings. The use of agonistic anti-CD40 antibodies as a single intervention has been successful at activating tumor DCs to stimulate T cell rejection of a murine tumor model (109). In addition, a fully human CD40 agonist antibody, CP-870,893, has been tested in humans with advanced cancers, resulting in objective responses in 14% of patients (110). However, mechanistic studies in preclinical models identified macrophages as direct mediators of cytolytic anti-tumor activity, with negligible contribution of anti-tumor T cells (111). Another trial using weekly dosing of CP-870,893 in advanced cancer patients showed only stable disease as the best clinical response. Some patients showed decreased T cell numbers, indicating that the dosing interval may have been too frequent (112). In our preclinical systems or in human tumor-derived DCs, CD40 agonists alone had no measurable effect on DC activation *in vivo* (34). However, based on the optimization of multiple combinations of vaccine adjuvants carried out by Ahonen et al. (113), we confirmed that CD40 and TLR3 agonists synergize to transform ovarian cancer-associated DCs from an immunosuppressive to an immunostimulatory cell type, both *in vivo* and *in situ* (34). These findings indicate the importance of acknowledging differences in the TME among various cancers, including that treatments like CD40 agonists may act on cells other than DCs. While in some tumors, CD40 agonists may singlehandedly activate DCs, in other tumors, like ovarian carcinoma, combining CD40 agonists with TLR activation will be necessary to revert DCs from immune-suppressors into functional APCs.

The other component of our synergistic combination, TLR agonists, can also activate DCs and has been tested in cancer with some success as a monotherapy. TLR9 agonists, for instance, have been developed and are in clinical trials (114). Stronger stimulation of DCs can be achieved through the activation of TLR3 with poly(I:C). To address its undesirable toxicity in humans, a less stable version of poly(I:C) called poly(I:C_12_U) was developed by incorporating a mismatched uracil, which still functionally activates DCs and enhances their IL-12 production (115).

Recent studies from our group have also underscored the potential of immunostimulatory nanoparticles carrying functional RNA against tumors that are compartmentalized, such as ovarian cancer. We showed that nanocomplexes comprised of polyethylenimine (PEI), a biocompatible polymer and TLR5 agonist, and siRNA oligonucleotides targeting PD-L1 are selectively engulfed by DCs in the ovarian cancer microenvironment (32). Activation of multiple TLRs and PD-L1 silencing synergize to promote the capacity of DCs at tumor locations to present the tumor antigens that they spontaneously phagocytose in the TME. Taking advantage of this optimized system, we were also able to deliver immunostimulatory miR-155 specifically to immunosuppressive DCs in mouse ovarian tumors, through the use of synthetic, functional double-stranded RNA Dicer substrates (37). Augmenting miR-155 activity resulted in genome-wide transcriptional changes that transformed DCs from a regulatory to an immunostimulatory phenotype. Although human tumor ascites primarily accumulates canonical macrophages, it is plausible that miR-155 supplementation also exposes their capacity to effectively present antigens. In addition, DCs are recruited to solid ovarian cancer masses, where they accumulate at the growing edge, in contact with ascites. Intra-peritoneal delivery of immunostimulatory nanocomplexes, therefore, offers significant promise to reverse the immunosuppressive activity of ovarian cancer-associated phagocytes. These approaches, however, need to be clinically tested.

Finally, one method that harnesses the power of DCs is actually a beneficial side effect of a classic treatment: chemotherapy. Certain chemotherapies are now understood to cause an immunogenic death of tumor cells that primes DCs to activate an anti-tumor immune response. This mechanism was first described for doxorubicin, showing that it induced immunogenic, caspase-driven tumor cell death that stimulated a protective immune response dependent on DCs and CD8 T cells (116). The features of immunogenic cell death include the release of ATP (117), surface exposure of calreticulin (118), and secretion of HMGB (119), which respectively act to recruit (120), induce engulfment by (121), and activate DCs for T cell stimulation. The list of agents inducing immunogenic cell death has been extended to include anthracyclines, oxaliplatin (but not cisplatin), and irradiation, among others. For a recent comprehensive review, the reader is referred to Kroemer et al. (122).

## Final Remarks

Pathological myelopoiesis in cancer individuals results in the accumulation of a heterogeneous mix of MDSCs, macrophages, immature DCs, and regulatory DCs. This results in defective antigen presentation, which causes T cell anergy and, especially, exhaustion. In addition, certain tumors mobilize classical DCs with immunosuppressive activity known as regulatory DCs. All these mechanisms have hindered the success of DC-based vaccines. However, novel approaches aiming to prevent tumor recurrences at early stages or using DCs for *ex vivo* priming of tumor-reactive lymphocytes offer significant promise. Finally, the antigen-presenting capacity of tumor-infiltrating DCs can be promoted *in vivo* and *in situ*, thus achieving the double goal of reversing immunosuppression and directly boosting protective immunity.

## Conflict of Interest Statement

The authors declare that the research was conducted in the absence of any commercial or financial relationships that could be construed as a potential conflict of interest.
